# In vitro fermentation and camellia oil emulsification characteristics of konjac glucomannan octenyl succinate

**DOI:** 10.1002/fsn3.1702

**Published:** 2020-06-08

**Authors:** Fan‐Bing Meng, Qian Zhang, Yun‐Cheng Li, Shu‐Yan Liu, Da‐Yu Liu, Hua Yu

**Affiliations:** ^1^ College of Pharmacy and Biological Engineering Chengdu University Chengdu China; ^2^ Key Laboratory of Coarse Cereal Processing Ministry of Agriculture Chengdu China

**Keywords:** camellia oil, in vitro fermentation, konjac glucomannan octenyl succinate, nanoemulsion, stabilization

## Abstract

It is important to select an appropriate emulsifier to overcome the poor stability and dispersibility of the vegetable oils in food system. Previous studies suggest that OSA‐modified konjac glucomannan (KGOS) has potential to be used as a food emulsifier. In this study, in vitro fermentation suggested that KGOS could promote the growth of the important intestinal probiotics *Lactobacillus* and *Bifidobacterium* and then promote intestinal fermentation to produce gas and short chain fatty acids. The emulsification experiments indicated that KGOS had good emulsification ability and stability for camellia oil. Under 40 MPa for 90 s homogenization, 0.2% (w/w) KGOS could encapsulate 20% (w/w) camellia oil. The nanoemulsion was stable at a low pH and high concentration of NaCl and ethanol. Konjac glucomannan octenyl succinate encapsulation could prevent the oxidation of camellia oil at 25°C and storage for 30 days.

## Graphical abstract

KGOS could promote the growth of the important intestinal probiotics. KGOS/camellia oil nanoemulsion was stable at a low pH and high concentration of NaCl and ethanol. KGOS encapsulation could prevent camellia oil oxidation at 25°C and storage for 30 days.

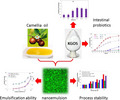

## 1 INTRODUCTION

Edible vegetable oils are commonly used in food systems because they are rich in bioactive ingredients, such as unsaturated fatty acids, phenolic compounds, and vitamin E (Lan et al., 2017; Sramala, Pinket, Pongwan, Jarussophon, & Kasemwong, 2016). Although these ingredients have many benefits for human health, they are very sensitive to heat, oxygen, and light during processing and storage, resulting in a potential alteration in nutritional composition and food quality (Lan et al., 2017). Moreover, in food processing, the vegetable oils are required to be continuously dispersed in food systems. Emulsification systems are usually adopted to overcome the poor stability and improve the dispersibility of the vegetable oils in a food system (McClements, 2010; Sramala et al., 2016; Yang, Guo, Sun, & Wang, 2016). Because nanoemulsions have many advantages, such as increased water dispersion and bioavailability, improved stability, and weakened scattering of light, due to their smaller droplet sizes (<200 nm) (Dos Santos, Andrade, Flores, & Rios, 2018; Troncoso, Aguilera, & Mcclements, 2012), they are commonly used to disperse and protect liposoluble food constituents. However, the processing and storage stability of edible vegetable oils need to be protected by large surfactants and cosurfactants, which has limited their application (Dos Santos et al., 2018). Moreover, the emulsifier type has significant effects on the oxidation stability of oil in an emulsion; some could even promote the oxidation of oils (Bush, Stevenson, & Lane, 2017; Chatterjee & Judeh, 2017). Therefore, it is very important to select an appropriate emulsifier during the preparation of edible vegetable oil nanoemulsions.

Among the nanoemulsion wall materials, octenyl succinic anhydride (OSA)‐modified polysaccharide has widely been applied in vegetable oil emulsification, due to its excellent emulsifying properties, renewability, and low cost (Li et al., 2018; Paramita, Furuta, & Yoshii, 2012; Varona, Martin, & Cocero, 2009). Specifically, commercial OSA‐modified starch (OSAS) has been widely applied in liposoluble ingredient emulsification in food systems (Baranauskiene, Rutkaite, Peciulyte, Kazernaviciute, & Venskutonis, 2016; Sharif et al., 2017; Yang et al., 2016). Through emulsification with OSA starch, black cumin essential oil showed stability against phase separation and coalescence, as well as persistent bacteriostatic properties, during 4 weeks storage at room temperature (Sharif et al., 2017). In our previous studies, OSA‐modified konjac glucomannan (KGOS) was prepared. The emulsification and target delivery test indicated that KGOS had better emulsification capacity and stability than OSAS and great potential to be used as a food emulsifier, stabilizer, and microcapsule wall material (Li et al., 2018; Meng, Li, Liu, Zhong, & Guo, 2018; Meng, Zheng, Wang, Liang, & Zhong, 2014; Zhong et al., 2018). However, the physiological effects of KGOS consumption on the intestinal environment and emulsification characteristics of KGOS as a vegetable oil emulsifier have not been explored.

Camellia oil is one of the popular edible vegetable oils in China. It is rich in polyunsaturated fatty acids (Luo et al., 2019; Zhu et al., 2019) and bioactive ingredients, such as vitamin E, sterols, squalene, and flavonoids (Zhu et al., 2019), and thus commonly applied in the food and cosmetics industries. The application of camellia oil requires emulsification to increase the stability and water dispersibility during processing and storage. Therefore, in this study, camellia oil was chosen to investigate the emulsification and stabilization of KGOS. The aim of this study was to further verify the emulsification ability and stability of KGOS. The results of this study could also provide a reference for industrial application of KGOS in edible vegetable oil stabilization.

## 2 MATERIALS AND METHODS

### 2.1 Materials

Konjac glucomannan (KGM, 95.0% purity) was obtained from the Konjac Association of the Chinese Society for Horticultural Science. Camellia oil was purchased from Hunan Jinhao Camellia Oil Co., Ltd. 2‐Octen‐1‐ylsuccinic anhydride (99.0% purity) was purchased from Sigma Chemical Co. (St. Louis, MO, USA). Kunming (KM) mice were purchased from Tengxin Biotechnology Co., Ltd, and all experiments conformed to the China Ministry of Science and Technology Guide for the Care and Use of Laboratory Animals. All chemicals were analytical grade unless otherwise stated.

### 2.2 Preparation of KGOS

Konjac glucomannan octenyl succinate was prepared using a microwave method described previously (Meng et al., 2014). Twenty grams of KGM and 2% (w/w) Na_2_CO_3_ were added to the reaction vessel. After homogenization, 20.00 g of ethanol solution (30%) was added slowly with agitation, and then 3% OSA (in proportion to KGM, w/w) was added. The mixture was placed in the MASII microwave reactor (Shanghai Sineo Microwave Chemistry Technology Co.) and heated at 300 W and 70°C for 20 min and then cooled to 25°C and blended with 40.0 ml of 30% ethanol solution for 5 min. The pH of the solution was adjusted to 6.5 with HCl solution (1 N). The mixture was filtered and then washed with 30% ethanol five times to remove residual NaCl and other soluble impurities and with absolute ethanol to remove residual OSA. The final solid material was dried at 40°C for 24 hr and passed through a 100‐mesh nylon sieve. The substitution rate (SR) of KGOS was approximately 1.54%.

### 2.3 In vitro fermentation

In order to investigate the physiological effects of KGOS consumption on the intestinal environment, the samples were fermented in vitro under strict anaerobic conditions according to the method described by Goñi & Martin‐Carrón (1998): A fecal suspension was prepared from the cecal contents of five male KM rats with a ninefold normal saline dilution. A trace element solution was prepared with 132 g/L CaCl_2_·2H_2_O, 100 g/L MnSO_4_·4H_2_O, and 80 g/L FeC1_3_·6H_2_O; a macroelement solution was prepared with 9.45 g/L Na_2_HPO_4_, 6.2 g/L KH_2_PO_4_, and 0.6 g/L MgSO_4_·7H_2_O; a buffer solution was prepared with 4 g/L (NH_4_)HCO_3_ and 35 g/L NaHCO_3_. The fermentation medium contained 0.1 ml of trace element solution, 200 ml of macroelement solution, 200 ml of buffer solution, 1 g of Tryptone, 1 g of yeast extract, and 500 ml of distilled water. The fermentation medium was sterilized at 121°C for 15 min.

The in vitro fermentation was performed in 100‐mL piston‐type sealed culture tubes. The fermentation medium contained 10% fecal suspension and substrates. The substrates were set as follows: KGOS addition group (K1: 0.25% KGOS, K2: 0.5% KGOS, K3: 1% KGOS), positive control group (K + G: 0.5% KGOS + 0.5% glucose, G: 1% glucose), and negative control group (N: no KGOS or glucose addition). The fermentation was performed at 37°C for 24 hr, and then, the fermentation was stopped by using 75 μl of the 20 g/L CuSO_4_ solution. The gas production was directly recorded at a fermentation time of 0, 2, 4, 6, 8, 10, 12, 14, 16, and 24 hr. Five milliliters of the medium at the end of fermentation was drawn to measure the pH.

### 2.4 Short chain fatty acid (SCFA) analyses

Analysis of the short chain fatty acid (SCFA) content in collected samples was determined by gas chromatography (GC‐2010, Shimadzu). Five milliliters of the medium at the end of fermentation was centrifuged at 1,000*g* for 5 min, and the supernatant was filtered through a 0.2 μm membrane. Two milliliters of filtrate was mixed with 200 μl of 50% sulfuric acid and 1 ml of anhydrous ethyl ether. After storage in 4°C for 1 hr, 1 μl of the upper organic solvent phase was pipetted to measure the SCFA content by using GC (GC‐2010, Shimadzu) equipped with a flame ionization detector and an Rtx‐wax column (30 m × 0.25 mm, 0.25 µm). The carrier gas was He at a column flow rate of 10.7 ml/min with a split ratio of 8:9, and the column temperature was 90°C.

### 2.5 Isolation and identification of microorganisms

The fermentation medium was gradually diluted to 10^−7^, and 100 μl of the samples of 10^−5^, 10^−6^, and 10^−7^ was cultured to count microorganism colonies. *Enterobacter* was cultured in EMB plate medium at 37°C for 24 hr; *Bifidobacterium* and *Lactobacillus* were anaerobically cultured in BBL and MRS plate media, respectively, at 37°C for 48 hr. The microbe counts were presented as lg(CFU/g).

### 2.6 Preparation of KGOS/camellia oil nanoemulsion

After the KGOS was dissolved in ultrapure water, 20% (w/w) camellia oil was added and homogenized using a GJB30‐40 high‐pressure homogenizer (Hangzhou Huihe Machinery Equipment Co., Ltd.). The effect of homogenization pressure (20, 30, and 40 MPa) and time (30, 60, 120, 150, 180, 210, and 240 s) on the particle size of the nanoemulsion was investigated. The particle size and polydispersity index (PDI) of the nanoemulsions were measured based on dynamic light scattering (DLS) measurements made using a Zetasizer Nano ZS90 instrument (Malvern Instruments Ltd, Malvern, UK) equipped with a He‐Ne laser (633 nm) and 90° collecting optics (Li et al., 2018). A FV1200MPE/FV1200 confocal scanning laser microscope (CSLM) (Olympus, Tokyo, Japan) equipped with a UPLANSAPO 100×/1.40 oil immersion objective lens was used to visualize the microstructure of the KGOS emulsions (20% oil) obtained by high‐speed homogenization (6,000 r/min, 2 min) and high‐pressure homogenization (40 MPa, 90 s). After one week of storage at room temperature, 1 ml of the samples was transferred to a 2 ml centrifuge tube, and then, 50 μl of 0.5% Nile blue A reagent prepared in isopropyl alcohol was added and mixed well by a Vortex‐Genie 2 mixer (Scientific Industries). Two microliters of the stained emulsion was drawn on a special slide and observed under the confocal scanning laser microscope having a Helium–Neon laser (HeNe) with excitation at 633 nm (Meng et al., 2018).

### 2.7 Effects of camellia oil and KGOS content on the nanoemulsion stability

In order to investigate the effects of camellia oil content on the emulsion stability, the KGOS/camellia oil nanoemulsions containing 0.2% (w/w) KGOS and 10%, 20%, 30%, 40%, and 50% (w/w) camellia oil were prepared. The particle size and PDI were measured at the initial preparation time and after 30 days of storage at room temperature.

In order to investigate the effects of the KGOS content on the emulsion stability, the KGOS/camellia oil nanoemulsions containing 20% (w/w) camellia oil and 0.2%, 0.3%, 0.4%, 0.5%, and 0.6% KGOS were prepared. The particle size and PDI were measured at the initial preparation time and after 30 days of storage at room temperature. In addition, the apparent viscosity and emulsification yield were also measured to judge the emulsion stability. Apparent viscosity was measured by using a rotating viscometer (NDJ‐5S). The emulsification yield (Ey) was measured according to the description by Kim and Morr (1996) with minor revision: A portion of the nanoemulsion and 300 ml of distilled water were added to a 500 ml round‐bottom flask. After mixing, the mixture was steam distilled for 2 hr. When the height of the oil column was constant, the oil volume “V” was recorded, and the Ey was calculated as follows:

$$\text{Ey (\textbackslash{}\% )}=\frac{V\times \rho }{m\times c}\times 100$$

where m is the quality of the emulsion, V is the volume of the oil phase after distillation, *ρ* is the density of camellia oil, and c is the mass fraction of theamellia oil in the emulsion. The oil retention rate (RR, %) of the camellia oil after storage for 15 days and 30 days was calculated as follows:

$$\text{RR (\textbackslash{}\% )}=\frac{\text{Ey of camellia oil after storage}}{\text{Ey of camellia oil in initially sample}}\times 100.$$

### 2.8 Effects of pH, NaCl, and ethanol on nanoemulsion stability

A certain amount of KGOS was dispersed in deionized water to obtain a 0.4% (w/w) dispersion. Twenty percent (w/w) camellia oil was added to the dispersions, and the nanoemulsions were prepared by the method described above. Thirty milliliters of the nanoemulsion was used to evaluate the effects of pH (1, 2, 3, 4, 5, 6, 7, 8, 9, 10, 11, 12, and 13), NaCl (3.33, 6.67, 10.0, 13.33, and 16.67 mol/L), and ethanol (1%, 2%, 3%, 4%, and 5%) on the emulsion stability. The particle size and PDI were measured at the initial preparation time and after 30 days of storage at room temperature.

### 2.9 Effects of storage conditions on nanoemulsion stability

A certain amount of the nanoemulsion and unemulsified camellia oil were weighed for vacuum packaging or nonvacuum packaging and then stored at 4°C or 25°C either in shaded conditions or exposed to light. The camellia oil concentrations were measured every ten days according to the method described by Chaiyasit, Silvestre, McClements, and Decker (2000) with minor revision: A certain amount of the stored nanoemulsions or camellia oil was added to 2 ml of isooctane/2‐propanone (3:2; v/v) and then vortexed three times for 10 s each. The extraction solution was centrifuged for 2 min at 2000 g, and the clear upper layer was pipetted and was mixed with 4.5 ml of methanol/1‐butanol (2:1; v/v) and 30 μl of thiocyanate/Fe^2+^. After 20 min of incubation at room temperature, the absorbance was measured at 510 nm. The lipid peroxide concentrations were quantified by using a cumene hydroperoxide standard curve.

### 2.10 Statistical analysis

All tests were performed in triplicate, and the results are expressed as the mean ± standard deviation. SPSS 20.0 was used to analyze the data. Analysis of variance (ANOVA) was performed to determine the significance at *p* < .05 by Tukey's HSD test.

## 3 RESULTS AND DISCUSSION

### 3.1 The effect of KGOS on the gas production and pH changes

**FIGURE 1:**
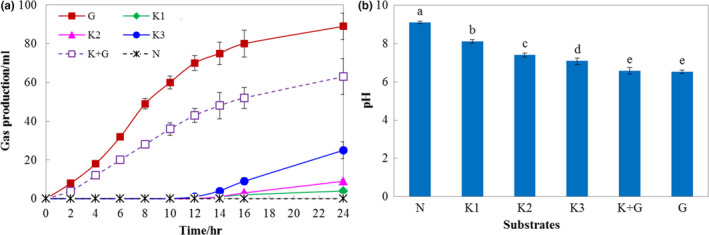
Effects of different samples on gas production (a) and pH (b) during in vitro fermentation. K1: 0.25% KGOS, K2: 0.5% KGOS, K3: 1% KGOS, K + G: 0.5% KGOS + 0.5% glucose, G: 1% glucose, N: no KGOS or glucose addition. Values with different superscripted lowercase letters are significantly different (*p* < .05) using Tukey's HSD test

KGM, which has served as a prebiotic (Zhao & Geng, 2016), is a soluble dietary fiber to improve carbohydrate metabolism, bowel movement, and colonic ecology (Chua, Baldwin, Hocking, & Chan, 2010). The prebiotic substance could promote intestinal microbiota fermentation to produce gas and short chain fatty acids (SCFAs), which can result in a lowered colonic pH (Erickson, Carlson, Stewart, & Slavin, 2018). The results of Figure 1 indicated that the OSA‐modified konjac glucomannan (KGOS) also showed fermentable characteristics. Especially after 12 hr of fermentation, the gas production increased with the increase in KGOS concentration. However, compared to that in the group with glucose addition, the gas production of the KGOS addition group was significantly lower, which indicated that the KGOS slowly decomposed during fermentation (Figure 1a). The pH changes also verified this speculation. The pH of the fermentation broth significantly (*p* < .05) decreased with the increase in KGOS concentration (Figure 1b). However, the gas production and pH change in the K + G group showed no significant difference from the G group, which indicated that KGOS has less interference with intestinal microorganisms than glucose.

### 3.2 The effect of KGOS on SCFAs production

**FIGURE 2:**
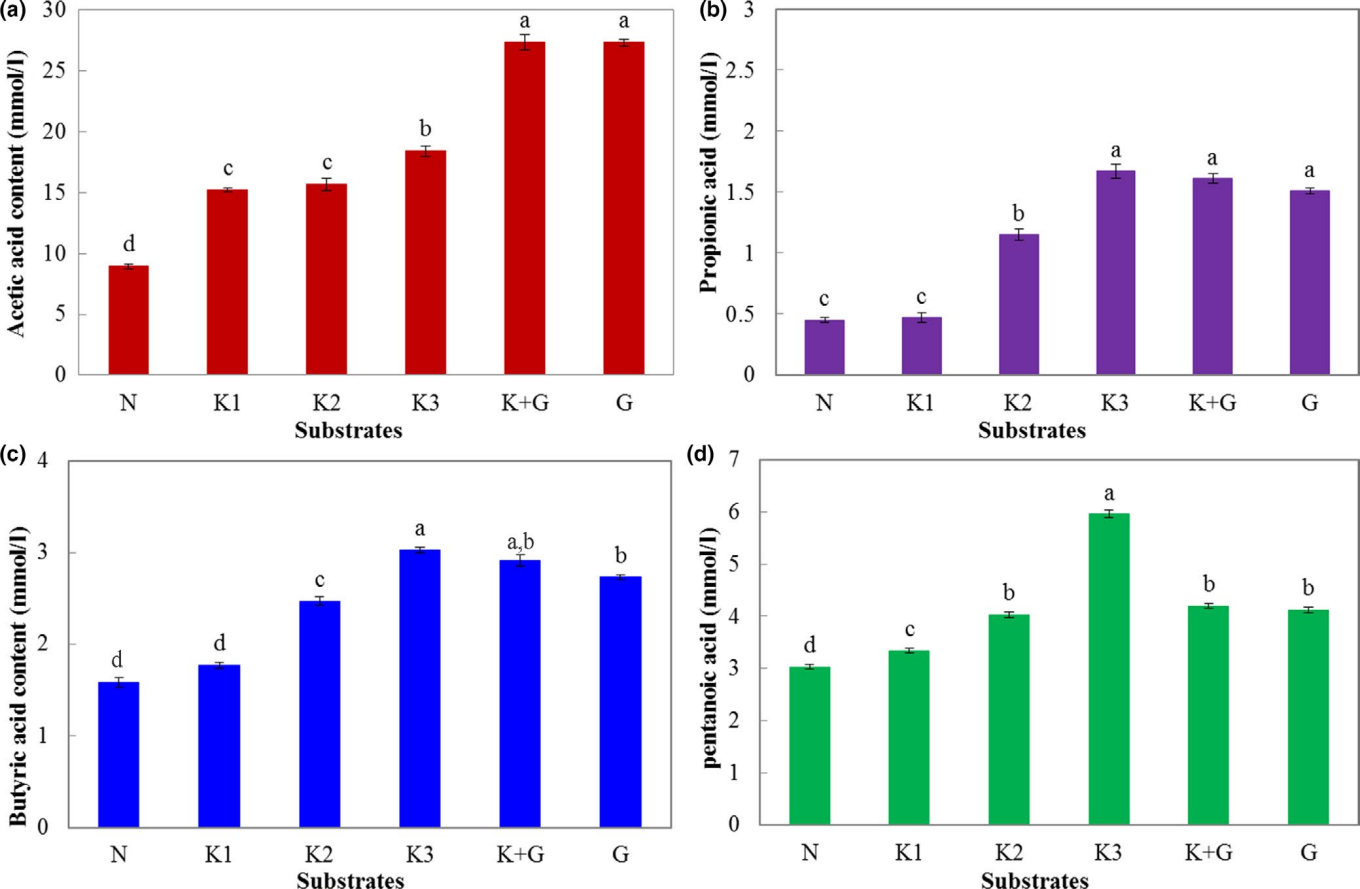
Effect of different samples on short chain fatty acid production during fermentation. K1: 0.25% KGOS, K2: 0.5% KGOS, K3: 1% KGOS, K + G: 0.5% KGOS + 0.5% glucose, G: 1% glucose, N: no KGOS or glucose addition. Values with different superscripted lowercase letters are significantly different (*p* < .05) using Tukey's HSD test

The effects of KGOS on SCFAs production are shown in Figure 2. The SCFAs production significantly increased (*p* < .05), except for the propionic acid production in the K1 group. When the KGOS concentration increased to 1%, the production of propionic acid, butyric acid, and pentanoic acid in the fermentation broth was significantly higher than that in the other groups. Moreover, compared to the G group, the SCFAs production of the K + G group was similar (acetic acid and pentanoic acid) or slightly decreased (propionic acid and butyric acid), which suggested that KGOS did not negatively affect the growth of intestinal microbiota. Colonic fermentation of dietary fibers by the intestinal microbiota will generate some organic acids including SCFAs (Chawla & Patil, 2010), especially propionic and butyric acids. Increasingly, more scientific evidence demonstrates that both propionic and butyric acids have desirable biological functions (Chua et al., 2010). Therefore, the increase in SCFAs production during fermentation also indicates that KGOS has the function of promoting intestinal prebiotics like KGM.

### 3.3 The effect of KGOS on the growth of intestinal microorganisms

**FIGURE 3:**
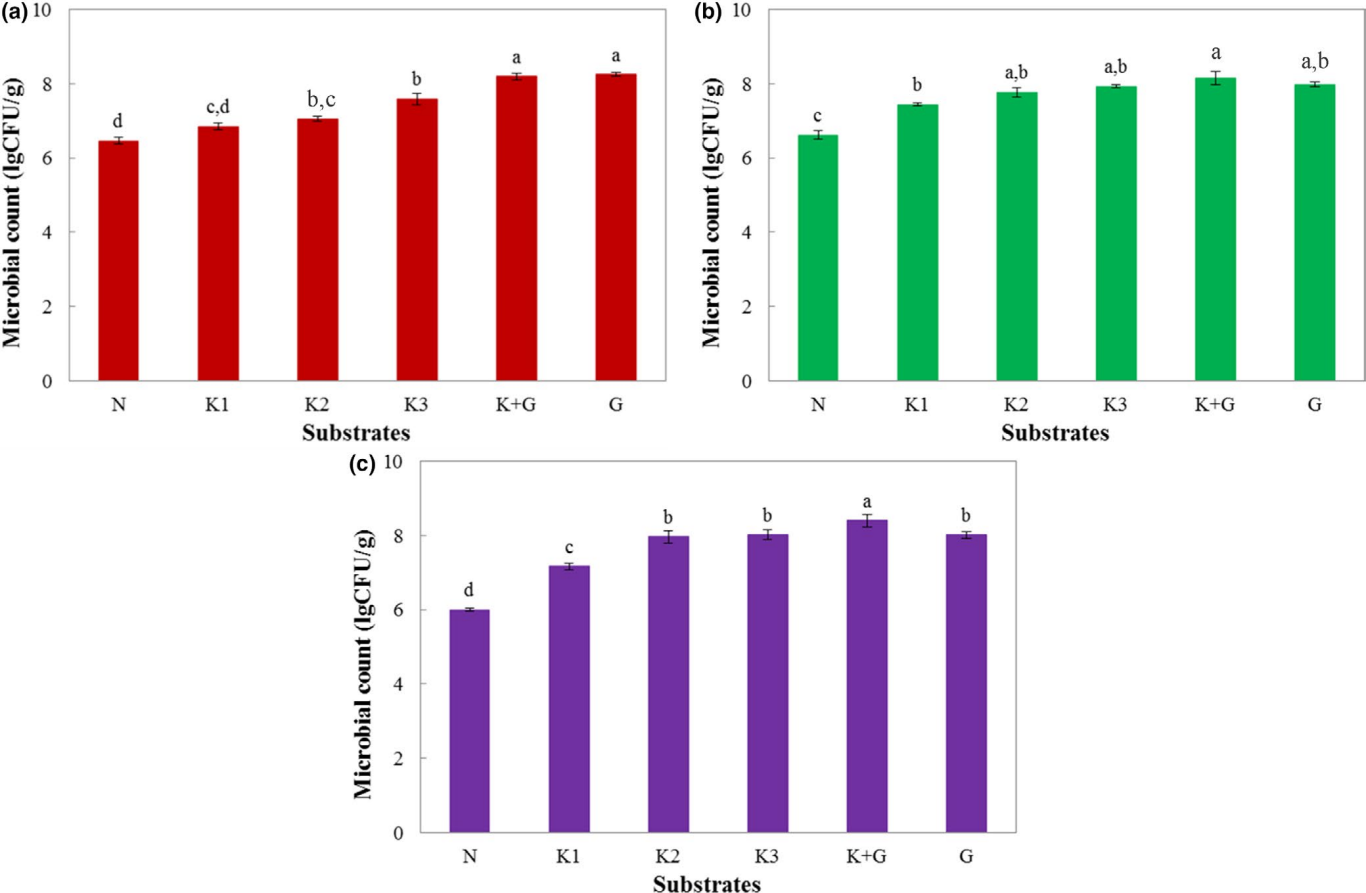
Effects of different substrates on *Enterobacter* (a)*, Lactobacillus* (b), and *Bifidobacterium* (c) during fermentation. K1: 0.25% KGOS, K2: 0.5% KGOS, K3: 1% KGOS, K + G: 0.5% KGOS + 0.5% glucose, G: 1% glucose, N: no KGOS or glucose addition. Values with different superscripted lowercase letters are significantly different (*p* < .05) using Tukey's HSD test

In order to further investigate how KGOS affects the intestinal microbiota, the microorganisms in the fermentation broth were isolated and identified. From the results in Figure 3, KGOS could promote the growth of three intestinal microorganism genera: *Enterobacter*, *Lactobacillus,* and *Bifidobacterium*. However, the promotion of growth by KGOS for *Lactobacillus* and *Bifidobacterium*, two important intestinal probiotics (Gonzalez‐Bermudez, Lopez‐Nicolas, Peso‐Echarri, Frontela‐Saseta, & Martinez‐Gracia, 2018), was greater than for *Enterobacter*, but glucose showed the opposite trends. This result also suggested that KGOS has certain intestinal probiotics as a food emulsifier. Previously, study (Chen, Cheng, Wu, Liu, & Liu, 2008) indicated that KGM could effectively increase the numbers of *Bifidobacteria*, *Lactobacilli,* and total bacteria in the daily fecal output, beneficially increase the relative proportions (% of total bacteria) of *Bifidobacteria* and *Lactobacilli*, and decreased the relative proportion of clostridia compared with the placebo. Besides, Harmayani, Aprilia, and Marsono (2014) reported that diet supplementation with KGM could inhibit the growth of *Escherichia coli*, enhance the production of total SCFA, and reduce pH value of cecal content, which indicated that KGM may be used as functional food. In total, KGM is a macromolecular compound for reaching the intestine and can be served as prebiotics (Zhao & Geng, 2016). From Figure 3, KGOS as a low‐substituted esterified derivative of KGM has also similar prebiotic functions with KGM.

### 3.4 Preparation of the KGOS/camellia oil nanoemulsion

**FIGURE 4:**
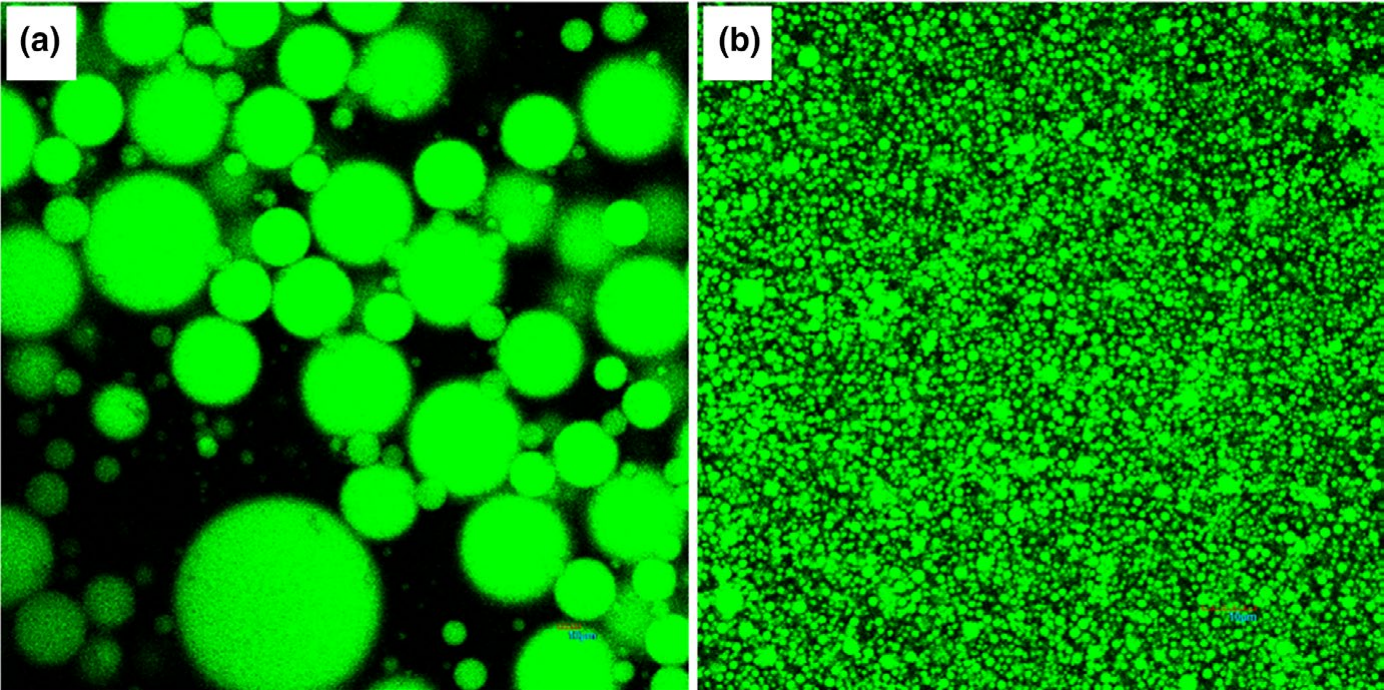
Microstructures of KGOS/camellia oil‐in‐water emulsions using CSLM. (a) Emulsion obtained by high‐speed homogenization, and (b) nanoemulsion obtained by high‐pressure homogenization

Camellia oil is rich in polyunsaturated fatty acids and bioactive ingredients, so the application of camellia oil requires emulsification to increase the stability and water dispersibility during processing and storage (Luo et al., 2019; Zhu et al., 2019). There are two common ways of emulsion formation: high‐speed homogenization and high‐pressure homogenization. From the CSLM observations shown in Figure 4, the particle size of the emulsion obtained by high‐speed homogenization varied widely and could not reach the nanometer scale (Figure 4a). After high‐pressure homogenization, the particle size of the emulsion reached the nanometer scale and was distributed uniformly (Figure 4b and Table 1).

**TABLE 1:** Effects of pressure and preparation time on nanoemulsion particle size and PDI

	60 s	90 s	120 s	150 s	180 s	210 s
20M						
Particle size (nm)	10.43 ± 0.34^a^	8.81 ± 0.521 ^ab^	9.91 ± 0.59^a^	9.25 ± 1.19^ab^	7.73 ± 0.08^ab^	6.41 ± 0.25^b^
PDI	0.57 ± 0.01^ab^	0.59 ± 0.04^a^	0.40 ± 0.03^c^	0.40 ± 0.06^c^	0.41 ± 0.00^c^	0.42 ± 0.02^bc^
30M						
Particle size (nm)	6.67 ± 0.49^a^	6.30 ± 0.25^a^	5.71 ± 0.21^ab^	4.93 ± 0.15^bc^	4.16 ± 0.07^bc^	4.22 ± 0.12^c^
PDI	0.43 ± 0.04^a^	0.36 ± 0.02^ab^	0.30 ± 0.01^b^	0.3 ± 0.02^b^	0.27 ± 0.01^b^	0.28 ± 0.01^b^
40M						
Particle size (nm)	3.16 ± 0.01^a^	2.48 ± 0.03^b^	2.40 ± 0.04^b^	2.39 ± 0.08^b^	2.46 ± 0.04^b^	2.33 ± 0.10^b^
PDI	0.25 ± 0.00^a^	0.27 ± 0.01^a^	0.25 ± 0.01^a^	0.25 ± 0.01^a^	0.24 ± 0.01^a^	0.26 ± 0.02^a^

Note

Values with different superscripts lowercase letters in same line are significantly different (*p* < .05) using Tukey's HSD test.

From the results in Table 1, after 60 s of high‐pressure homogenization at 20 MPa, the particle size of the KGOS/camellia oil nanoemulsion was 10.43 nm, and the particle size decreased with the increase in homogenization pressure and time. When the pressure increased to 40 MPa, the particle size and PDI of the nanoemulsion had no significant difference. In order to avoid a sharp rise in temperature caused by increased pressure, a homogenization pressure of 40 MPa and preparation time of 90 s were selected for the following experiment.

### 3.5 Effects of camellia oil and KGOS content on the nanoemulsion stability

**FIGURE 5:**
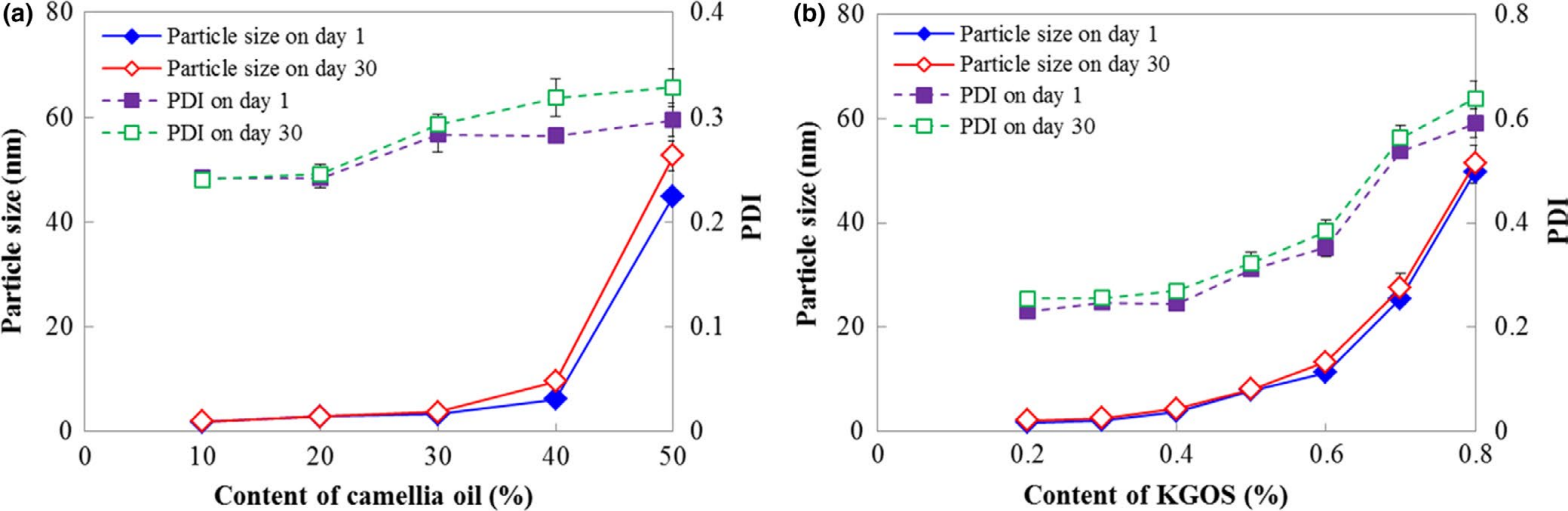
Effects of the camellia oil content (a) and KGOS content (b) on the stability of the nanoemulsion

The oil concentration had an effect on nanoemulsion stability during storage. When the camellia oil concentration was greater than 40% (w/w), the particle size of the nanoemulsion increased significantly during 30 days of storage (Figure 5a). The KGOS content also affected the nanoemulsion stability. A low KGOS content showed slight effects, but when the KGOS content was greater than 0.5% (w/w), the particle size and PDI increased significantly with the increasing of KGOS content. This finding was probably because KGOS is a macromolecule surfactant and the homogeneity of the particle size is poor. The molecular envelope increased with an increase in KGOS content. However, high pressure makes intermolecular entanglement very tight, so the nanoemulsion showed good storage stability. After 30 days of storage, the particle size of the nanoemulsion did not change significantly for all emulsions with different KGOS concentrations (Figure 5b).

**FIGURE 6:**
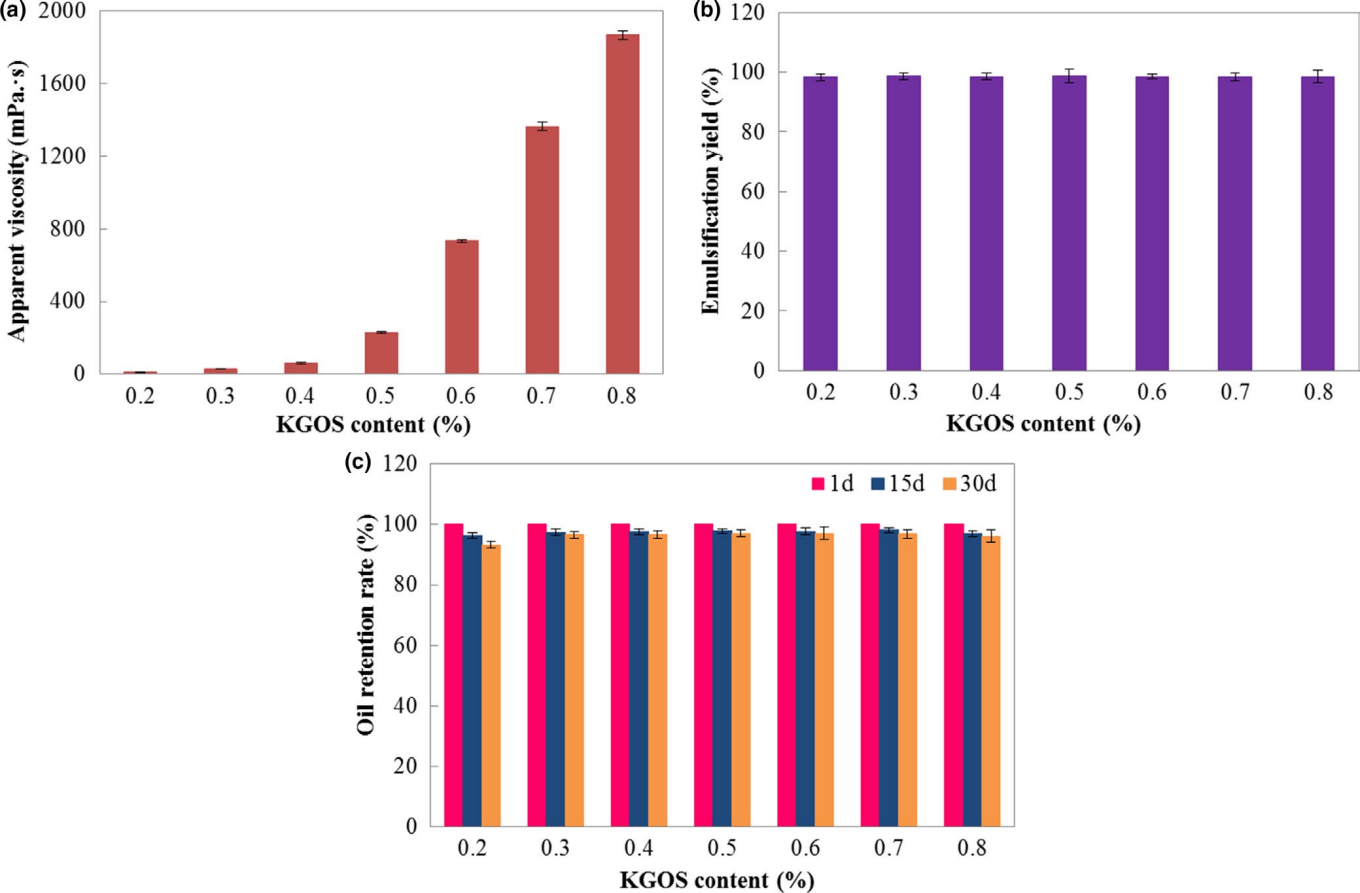
Effects of the KGOS content on the apparent viscosity (a), emulsification yield (b), and oil retention (c) of the nanoemulsion

Because KGOS has a strong thickening property like KGM (Li et al., 2018), the apparent viscosity of the KGOS/camellia oil‐in‐water nanoemulsions increased significantly with an increase in KGOS concentration (Figure 6a). When the KGOS content was 0.5% (w/w), the apparent viscosity was 230.67 mPa s, but when the KGOS content increased to 0.8%, the apparent viscosity significantly increased to 1868.02 mPa·s. The increase in the viscosity prolonged the homogenization time and brought difficulties to actual production. However, a low content of KGOS (lower than 0.5%) did not reduce the emulsification yield (Figure 6b), and the emulsification yield was always maintained at a high level (greater than 98%), even when the KGOS content was 0.2%. In addition, the KGOS/camellia oil‐in‐water nanoemulsions had good storage stability. After 30 days of storage, the camellia oil retention rate of the sample prepared by 0.2% KGOS content was 93.31%, and the other samples were all kept as high as 95% (Figure 6c). The emulsification yield and stability were similar to those in the report by Liang et al. (2012) which used 12% (w/w) succinylated waxy maize starch to emulsify peppermint oil, but a lower concentration of KGOS was used in our study. These results further suggested that KGOS has great potential in the preparation of edible vegetable oil nanoemulsions or in the use of microcapsule wall materials. Moreover, the viscosity of KGOS is higher than OSA‐modified starch (OSAS) (Meng et al., 2014), so it can be developed and utilized according to different viscosity requirements in practical application.

### 3.6 Effects of pH, NaCl, and ethanol on nanoemulsion stability

**FIGURE 7:**
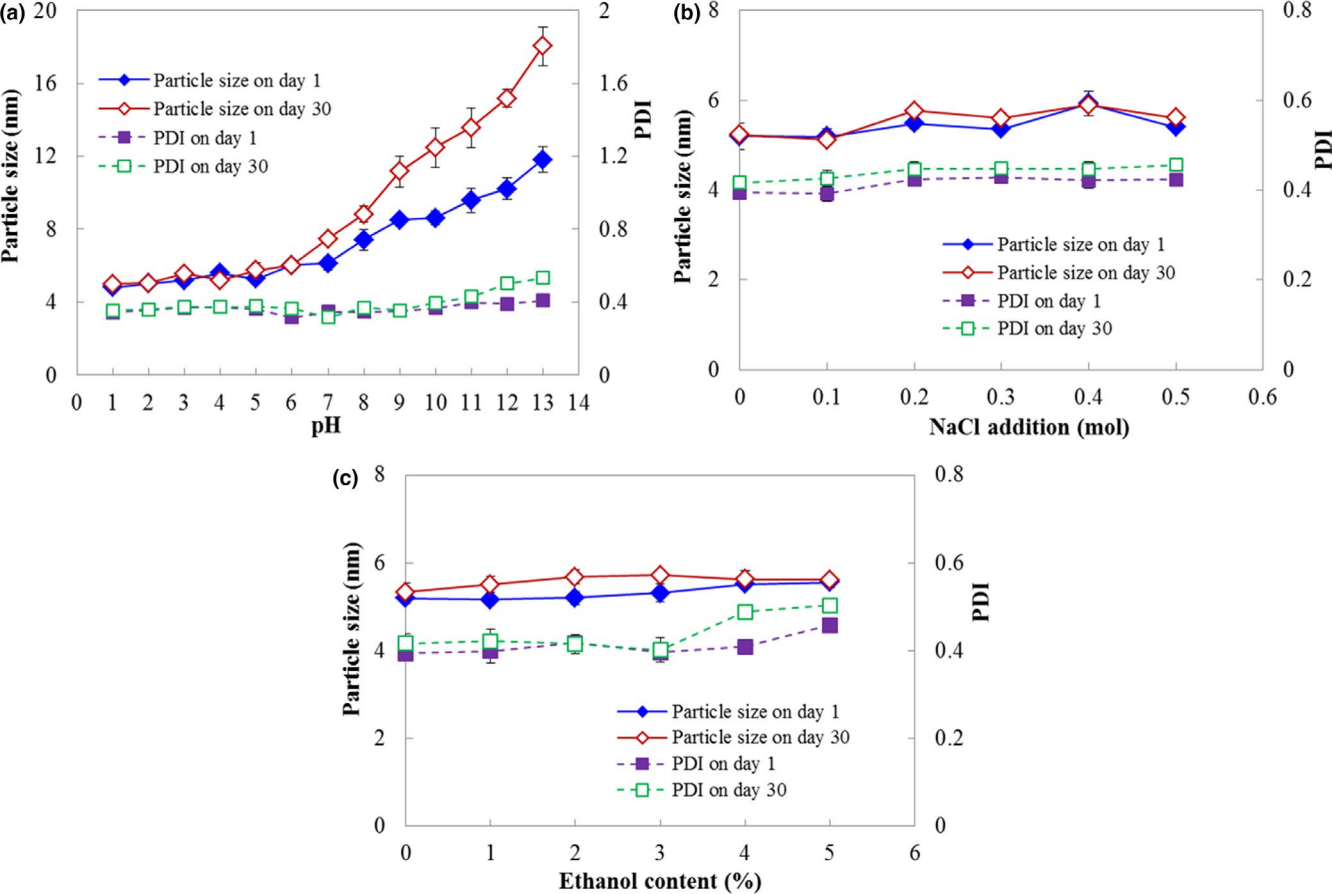
Effects of the pH (a), NaCl (b), and ethanol (c) on the particle size and PDI of the nanoemulsion

The stability of emulsions was affected by the change in pH, ionic composition, and some other small molecular compounds, such as ethanol, which led to de‐emulsification and leakage of the entrapped compounds (McClements, 2012; Tan et al., 2014). From the results in Figure 7a, the particle size and PDI of KGOS/camellia oil‐in‐water nanoemulsions were almost unchanged at low pH (≤6). These results might be because the amount of electrostatic charge on the surface of the KGOS emulsions is large under acidic conditions, so the electrostatic repulsion force between the droplets is large enough to keep the oil‐in‐water droplets in equilibrium and without aggregation, thus maintaining the stability of the nanoemulsion. However, when the pH was higher than 7, the particle size and PDI increased with an increase in pH, which indicated that the storage stability decreased. Under alkaline conditions, the acetyl groups on the KGOS chains are easily broken (Dave & McCarthy, 1997; Huang, Takahashi, Kobayashi, Kawase, & Nishinari, 2002), which makes the winding structure of the KGOS/camellia oil nanoemulsion unstable and de‐emulsified.

The effects of NaCl and ethanol on nanoemulsion stability are shown in Figure 7b,c. The addition of NaCl and ethanol did not significantly change the average particle size and PDI of the nanoemulsion, which indicated that KGOS, as a nonionic polymer surfactant, shows excellent performance in the presence of these components and has potential application value in the food industry.

### 3.7 Effects of storage conditions on nanoemulsion stability

**FIGURE 8:**
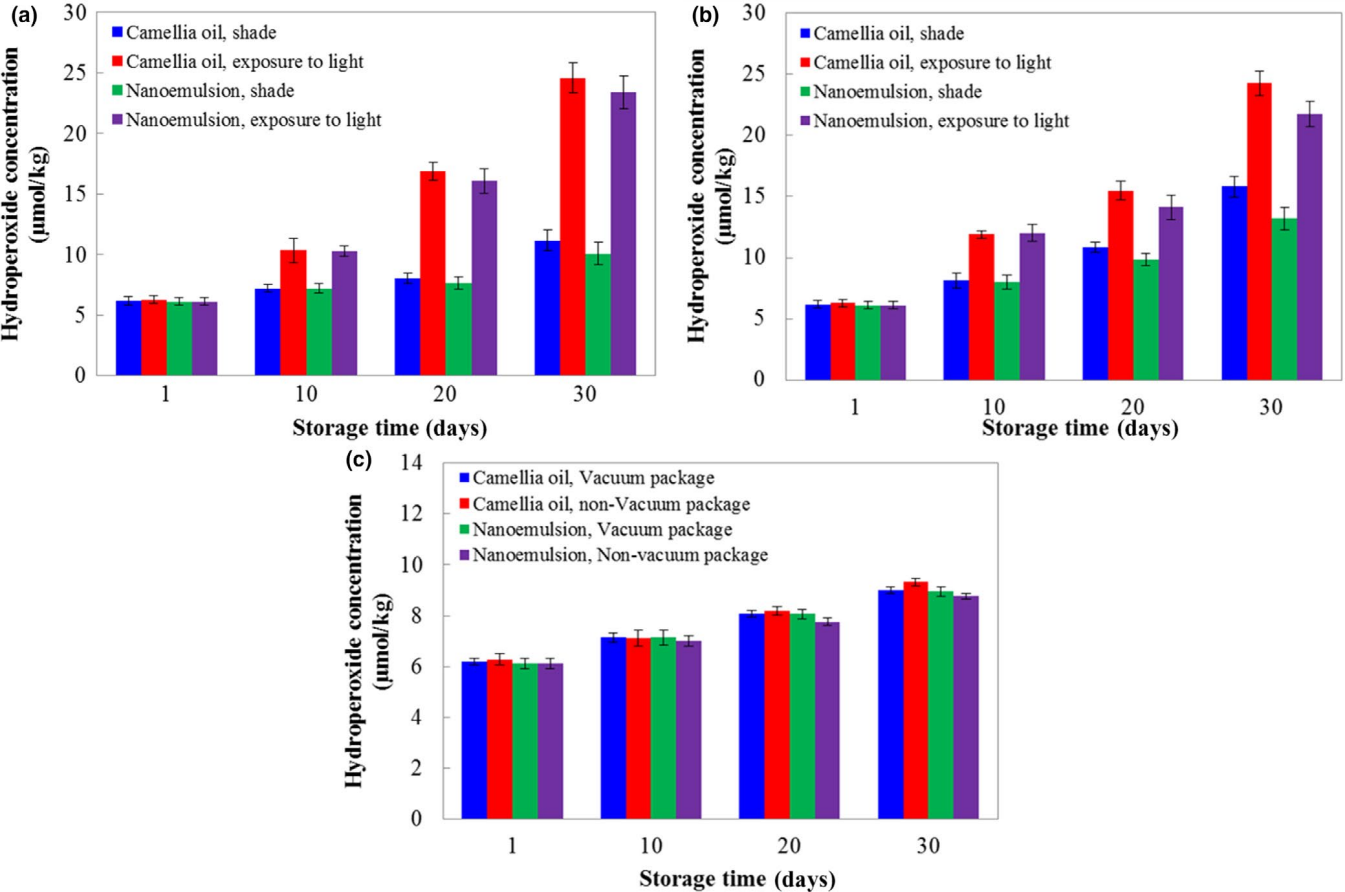
Effects of storage conditions on the hydroperoxide concentration of camellia oil. (a) Vacuum packaged and stored at 25°C, and (b) nonvacuum packaged and stored at 25°C and (c) stored at 4°C with shade

The effects of storage conditions on nanoemulsion stability were measured by the hydroperoxide production of camellia oil. From the results shown in Figure 8, light had a large influence on the yield of hydroperoxide. With the prolongation of storage time, the content of hydroperoxide in the samples under shaded conditions was significantly lower than that of the samples exposed to light. KGOS encapsulation prevented the oxidation of camellia oil. Under 25°C and shaded storage for 30 days, the hydroperoxide of nanoemulsions with vacuum and nonvacuum packaging was 9.7% and 16.4% lower, respectively, than that of unencapsulated camellia oil (Figure 8a,b), and under light exposure storage, the results were 4.9% and 10.2%, respectively (Figure 8a,b). Low temperature helps to protect oil from oxidation. The hydroperoxide production levels in all samples at 4°C were approximately 11 μmol/kg after 30 days of storage (Figure 8c).

The type of emulsifier greatly affected the oxidation stability of the oil‐in‐water emulsion, and some could even promote the oxidation of oils (Bush et al., 2017; Chatterjee & Judeh, 2017; Kasaikina et al., 1999) Ionic emulsifiers were easily affected by pH and salts, but neutral emulsifiers, especially for polysaccharides, could provide emulsion stability over wide pH ranges and various ionic strengths (Sivapratha & Sarkar, 2018), which are very important components in food systems. From the results of this study, as a type of polymer surfactant, KGOS had good emulsifying and stabilizing characteristics for camellia oil, so it has good potential application value in edible vegetable oil emulsification.

## 4 CONCLUSION

In conclusion, the in vitro fermentation experiments suggested that the octenyl succinic anhydride (OSA)‐modified konjac glucomannan (KGOS) did not negatively affect intestinal microorganisms but could promote the growth of the important intestinal probiotics *Lactobacillus* and *Bifidobacterium* and then promote intestinal fermentation to produce gas and short chain fatty acids (SCFAs). The emulsification experiments indicated that KGOS had good emulsification ability and stability for camellia oil. When the KGOS and camellia oil contents were 0.2% and 20% (w/w), respectively, the homogenization pressure and time were 40 MPa and 90 s, respectively. The emulsification yield of the KGOS/camellia oil nanoemulsion was greater than 98%, and the oil retention rate was higher than 93% after 30 days of storage. The particle size and PDI of the KGOS/camellia oil nanoemulsion were almost unchanged under acidic conditions, and the addition of NaCl and ethanol did not affect the storage stability. KGOS encapsulation could prevent the oxidation of camellia oil at 25°C and storage for 30 days.

## CONFLICT OF INTEREST

The author(s) declare no conflicts of interest.
